# Supplementary material for: Impact of pulmonary rehabilitation in sleep in COPD patients measured by actigraphy
Source: PLoS One. 2021 Mar 16;16(3):e0248466. doi: 10.1371/journal.pone.0248466 (PMC7963048; doi:10.1371/journal.pone.0248466)
Supplement: Attachment — Submitted filename: Response to Reviewr # 2.docx [file pone.0248466.s001.docx]

**PONE-D-20-23683**

**Response to Reviewer # 2**

Authors greatly appreciate the excellent review, comments, critics and recommendations by the reviewers. We have put forward our best effort to answer your concerns and comments and amended our manuscript as appropriate. We continue to value your critics and comments.

Here are our responses:

**1.** The authors state that they did a retrospective study. They analyzed a large dataset of subjects that attended the PR program at the VA system with COPD. My understanding is that the subjects had the actigraphy (all of them) for sleep assessment as a routine part of the program? Or to assess daily activity? Please explain. Was it planned initially as a research study? If not, please expand on why your program is using Actiwatch on a PR program.

**Response:** During early days of PR program at Loma Linda VA, a pilot study was done to incorporate activity tracker to track activity and sleep. The study included only few patients. An abstract was presented to ATS. However, the practice to use activity tracker continued as a standard of care for PR program at our institution.

No changes in manuscript made in response to this comment. Please advise if it is absolutely necessary amend the manuscript.

2. Expanding the discussion about male predominance is essential. The difference in sleep patterns from the gender perspective may help understand some of the results, and not limit the comment on the limitation section (which it should also be described as the authors did).

**Response:** added a paragraph:

Gender affects sleep as well. Females have reported poor quality of sleep, longer sleep latency and increased nighttime awakenings as compared to males[32], however, when measured by PSG, females have better sleep efficiency, more time spent sleeping and less awakening[33]. In elderly population, this gender difference may be very small or non-significant and females have better correlation between objective and subjective sleep qualities[34]. All these studies are done in healthy volunteers, therefore, gender effect in people with COPD has not been studied.

**3.** There is some discrepancy about the severity of the disease (please include 'severe and very severe' when describing the population -2 and 3- and the reference to GOLD severity guidelines). Why the patients had relatively preserved exercise tolerance measured by 6MW but are mostly on the 'severe' to 'very severe' category? That could happen, but less likely with such a high CAT score (mean of 24.8 +/- 7).

**Response:** Thank for pointing out the discrepancy. We added “very severe” in the sentence to reflect the accurate description of the severity of the disease.

We were also intrigued by the finding that exercise capacity was relatively preserved in our study population with severe or very severe COPD with very high CAT score. In my clinical practice, I obtain mMRC and CAT score on every patient on every visit. I also get 6MWT if there is any decline in functioning. For some reason, I have seen large discrepancy in mMRC and CAT score. I do not have large sample to cross validate this discrepancy with 6MWT in my practice. However, we know that there is a very good correlation between mMRC and CAT score (r=0.788, p<0.0001; Kaltsakas, ERJ 2013). We also know that dyspnea scales correlate with 6MWT. If I have to guess, I would suspect that it has to do something about discrepancy between various components of CAT score vs mMRC, especially components of CAT score asking about dyspnea. This is something I intend to pursue in a separate project. It is also difficult to evaluate the relative preservation of exercise in our population from this data set because of the nature of the study (we are studying 6MWT in a population who is expected to or are exercising).

4. As very briefly discussed by the authors, pharmacology may have played an essential role in this population. However, I didn't find the multivariate analysis considering this variable as a whole ('any meds that may affect sleep). The impact of such medications on sleep architecture, expanding the discussion, and running the analysis may help.

**Response**: This is very interesting aspect of COPD and sleep management and an intriguing question as well. We did consider COPD medications as a factor in our protocol and data analysis. We collected data on each category of the medications including short acting bronchodilators, LABA, LAMA and ICS. We also included other drugs affecting sleep such as sleep aids, psychoactive medications as well as pain medications. Nearly all participants were on short-acting bronchodilators (SABD) and nearly all were in some combination of LABA, LAMA or both. The difference in the pattern of SABD, LABA, LAMA and combination of those would be very small. Therefore, to isolate the effect of those medications on sleep on during and after PR, we would have required much larger sample. We did not track changes in COPD medication use pattern. Our PR program only admits patients after their medications were optimized and therefore, we do not expect large changes in COPD medications. To answer the effect of COPD medications on sleep and PR, a well-designed prospective study may be needed. For this reason, we did not even present data on COPD medications. In regards to all medications affecting sleep, we presented data on psychoactive medications and pain medications. Small proportions of participants were taking medications affecting sleep (less than 25% in all medication group affecting sleep).

No changes were made in response to this comment.

5. COPD and sleep quality have been assessed (objectively) with PSG, contrary to what the authors describe. Discussing differences/limitations on actigraphy on sleep assessment seems important, contributing to the negative results.

**Response:** We agree that Sleep in people with COPD has been objectively assessed by PSG. If the reviewer’s comment arise from the last paragraph of the “introduction” segment, we merely wanted to point out that there are paucity of data objectively evaluating effect of PR in sleep in people with COPD not in COPD population as such.

No changes were made to the manuscript in response to this comment.

6. The factors on the background influencing poor sleep (second paragraph) could use seminal references. The pharmacological effects and the reference (11) presented is not optimal.

**Response:** We agree seminal references would help the paper. We have added a reference to improve this.

*McCarthy, B., et al., Pulmonary rehabilitation for chronic obstructive pulmonary disease. Cochrane Database of Systematic Reviews, 2015(2).*

Spruit, M.A., et al., *An official American Thoracic Society/European Respiratory Society statement: key concepts and advances in pulmonary rehabilitation.* Am J Respir Crit Care Med, 2013. **188**(8): p. e13-64.

Ries, A.L., et al., *Pulmonary Rehabilitation: Joint ACCP/AACVPR Evidence-Based Clinical Practice Guidelines.* Chest, 2007. **131**(5 Suppl): p. 4S-42S.

We retained ref#11 for pharmacological effects since adding reference for individual agents would have been long.

7. The PR described (please add exercise tolerance on the third paragraph) also need some of the seminal or best references (Ries et al. in Annals, ATS PR document, or alike).

**Response:** Added exercise tolerance as recommended. Also added the following reference:

Ries, A.L., et al., *Pulmonary Rehabilitation: Joint ACCP/AACVPR Evidence-Based Clinical Practice Guidelines.* Chest, 2007. **131**(5 Suppl): p. 4S-42S.

8. The paragraph starting (background section) 'there is also evidence that COPD…….. outcomes' will fit better in the discussion section.

**Response:** Thank you for your careful review. If it is not prohibit, authors would like to keep the paragraph where it is. We believe it support the rationale why we wanted to do the study. We agree that it can fit well in the discussion section as well.

9. Do not abbreviate Loma Linda VA (VALL) as it is not standard.

**Response:** VALL has been removed and replaced with ‘VA Loma Lima Healthcare system’ or ‘our’ in the appropriate

10. Typo on the last paragraph on methods' Out' needs to be 'Our', I guess.

**Response:** Thank you for spotting the typographical error. I hunted down all “out” masquerading as “our”

11. Subheading explaining the actigraphy assessment. Briefly, what does, and how you use it (already described). Rephrase the 'use of the actigraphy' paragraph and put on the discussion. Also, it is simple to set, but not as simple to analyze for research purposes. A brief discussion of the actigraphy limitations seems necessary. "Actigraphy is a validated method to assess sleep, etc.'. It could be from Ancoli-Israel paper cited or equivalent.

**Response:** Added few sentences divided between method section and limitations. Hopefully it suffices.

An actigraph is small simple device typically worn on the wrist like a watch.  It can, however, be worn on an ankle or waist.  This device is a sophisticated movement sensor that continuously records movement and integrates it over time.  Mathematical algorithms are then utilized to estimate sleep and wakefulness time [17].

“Actigraphy has acceptable validity and reliability [19, 20, 49] but has several limitations as compared to PSG. Actigraphy is one dimensional measurement of sleep based on movements and PGS gathers sleep data from multiple inputs such as electroencephalogram, electro-oculogram and electromyogram[49]. Therefore, certain sleep variables such as SOL is more accurate in PSG[50].”

**12.** There is no clarity on the overweight group (BMI 25-<30). Please expand on the discussion as the mean was 29? Table result (27.5) and text do not match BMI; please correct with the right number.

**Response:** BMI group of less than 30, 30 to 39 and 40 or more was chosen for simplicity since half of the subjects in less than 30 BMI group had normal BMI. There was no significant difference between 3 BMI group (as above) or 4 BMI group (including normal, overweight, obese and morbidly obese) in regards to outcomes.

**The mean BMI was 29 and median BMI was 27.**5. Mean+/-SD presented in the abstract was changed to median BMI with IQR.

**13.** In the results section, limit to describe the % found. Avoid as much as possible 'nearly', majority, three-quarters by the %, etc. Just write the results.

**Response:** Those phrase were replaced by numbers/data where appropriate.

**14.** SGRQ has an error/typo; 31.1 or 61? See text/table discrepancy.

**Response:** Thank you for spotting this typographical error as well. I cross-checked and verified that the true value is 61.1. It was changed in the manuscript.

**15**. mMRC is not expected to change that much. The authors seemed surprised by the results. Personal comment.

**Response:** Thank you for sharing your experience and fact about mMRC. The truth is that I was surprised by the degree of change in mMRC.

16. The description of the cognitive findings can be on the table and expand on the discussion. Those are not the primary or secondary aim for the study.

**Response:** Stratified analysis by cognitive function and depression did not show any significant change in both objective and subjective sleep measures. Intuitively we expected some effect of mood or memory on sleep but we found none. Hence, we did not discuss futher. We have been collecting further data on short term and long term cognitive change after PR. We plan to discuss this issue further on next paper.

**17**. I would recommend running the analysis excluding patients on oxygen -26%- (improves sleep) and or obstructive sleep apnea (I assume there will be some). Both can interfere with the results. Despite having fewer subjects, may help better understand the results. It is very briefly discussed in two lines but a very general approach, and it is relevant for this study.

**Response:** We did perform stratified analysis based on oxygen use. Among people who were not using supplemental oxygen for chronic respiratory failure, there was no significant effect of PR on sleep (Actigraphy variables as well as PSQI).

Among people who used supplemental oxygen (n=18), total sleep time (TST) decreased marginally (pre-PR TST: 376±71 minutes, mean±SD, vs post-PR 321±100 minutes; p=0.02). Also, sleep efficiency improved marginally (pre-PR sleep efficiency: 76.0±8.4% vs post-PR sleep efficiency: 70.7±11.8%; p=0.03). Since the sample size was small, we did not report in the manuscript. Should the editor or reviewer believe that this information is significant for the manuscript, authors are ready to include and discuss in the full manuscript.

Similarly, stratified analysis of sleep variables (PSQI as well as actigraphy parameters) among people with or without sleep disorder, did not show any significant differences.

18. The results for PR are not necessary on the results section but a table and the discussion briefly (to demonstrate the program was effective). Are well known. Perhaps shortly name the ones that improved (quality of life, exercise tolerance, etc.).

**Response:** Authors belief that it improves the flow of reading. If it not prohibiti, authors would like to keep the paragraph in the Effect of PR on COPD related outcomes subheading.

19. The discussion could benefit from more profound thoughts from the authors regarding the benefits on the more severe patients (CAT score >20) as well as the ones with already low sleep quality (PSQI >5). Also, about the effects/impact of medication on sleep.

**Response:** In theory, we expected robust improvement in sleep variables in severe COPD patients (CAT score >20) and PSQI> 5. Stratified analysis did not show any benefit in sleep variables despite significant improvement in CAT score (among severe patient, CAT score >20: mean change – 6.32 points, 95% CI -4.4 to -8.3, p<0.001; not presented in the manuscript) after PR. Therefore, we did not want to speculate the effect or propose a theory based on our retrospective data.

20. Some brief discussion comparing objective measurements of sleep would help the reader not familiar with sleep medicine (PSG vs. Actigraphy).

**Response:** Added few sentences on Actigraphy in the method section and comparison in the limitation section. Details on PSG would have been long and confusing since none of our outcomes are based on PSG data.

21. The paragraph starting with 'Exercise is one of the ….' Has to be before the previous one 'exercise affects sleep.'

**Response**: Moved as recommended.

**22.** On the limitations, some paragraphs will be perhaps better to move up to the discussion section; 'Actigraphy has reasonable validity ….'. Also, the phrase is not too scientific; maybe it can be rephrased.

**Response:** Thank you for picking that up as well. I agree “reasonable” may be less scientific. However, society practice guidelines has described actigraphy as “acceptable”. Hence, will change to “acceptable” from “reasonable”

Morgenthaler, T., et al., *Practice parameters for the use of actigraphy in the assessment of sleep and sleep disorders: an update for 2007.* Sleep, 2007. **30**(4): p. 519-29.

**Recommendations:** Actigraphy provides an acceptably accurate estimate of sleep patterns in normal, healthy adult populations and inpatients suspected of certain sleep disorders.

23. Discussion about the patient population. This is relevant. And then briefly can be said in the limitation section as well.

**Response:** Added a paragraph:

Our cohort consistent of only Veterans and almost entirely males. Both of these factors can influence sleep. Veterans have high prevalence of psychiatric conditions such as PTSD, depression and anxiety as compared to general population [31]. Directly or indirectly these factors increase the prevalence of insomnia and poor quality sleep.

24. Nice tables. Perhaps the median doesn't add much to the study description. The dyspnea questionnaire expanded is not needed in much detail as it is beyond the study's intent. The Epworth was collected and had to be part of sleep discussion and assessment and not standing alone.

**Response:** Nearly half of the variable were normally distributed and rest were not. Representing data in both means+/-SD and median+/-IQR provides some symmetry. Also, expanded PFSDQ VAS score provides an additional measure for dyspnea. Authors would like to keep those measure if acceptable to the reviewers. If the reviewers truly believe that those should be truncated or removed, we will be happy to make changes.

In regards to ESS, added few sentences in the result section

ESS, which measures general sleepiness, was 8.0±5.1 (mean±SD) slightly higher than expected in normal individuals at baseline (ESS in normal individuals, 5.9±2.2, range 2-10)

The ESS did not change after PR in the entire cohort as well as when stratified by presence of absence of sleep disorders (stratified data not presented in the manuscript).

Since ESS was slightly above normal range and did not change in the overall cohort and in stratified analysis, we decided not to discuss it further.

Also, correction was made to the SD values for ESS in table 2 (the table contained SE not SD)

**25.** Perhaps the reader could benefit from a brief explanation of how sleep quality is measured. Many readers are not familiar with sleep variables.

**Response:** I did not quite understand how to respond to this comment. I added a small paragraph on PSQI in the method section. Discussion of all sleep variables would be too lengthy. I hope this suffices.

The added paragraph reads as below:

*PSQI measures sleep disturbances and sleep habits during last one month period. The questionnaire has 19 individual items and includes open ended questions and Likert-like items. It generates seven clinically derived domains or components of scores which includes: sleep quality, sleep latency, sleep duration, habitual sleep efficiency, sleep disturbances, use of sleeping medications, and daytime dysfunction. The global PSQI score is calculated by adding scores from all seven domains {Buysse, 1989}.*
