# Supplementary material for: Impact of pulmonary rehabilitation in sleep in COPD patients measured by actigraphy
Source: PLoS One. 2021 Mar 16;16(3):e0248466. doi: 10.1371/journal.pone.0248466 (PMC7963048; doi:10.1371/journal.pone.0248466)
Supplement: Attachment — Submitted filename: Response to the reviewer_2.docx [file pone.0248466.s002.docx]

**PONE-D-20-23683**

**Response to the reviewer**

**Comment from the reviewer:**

There are a number of discrepancies between data in tables and data in text.
For example
1) in the text: ”Seventy five percentage of the study population had a high or very high impact of COPD on their daily lives with a mean CAT score of 24.4 ± 7.1
2) in the table: CAT score, n=52, mean ± SD 24.8 ± 7.1

**Response:**

Thank you very much for spotting the typographical error. The value should be 24.8 ± 7.1 in the text as well (as in the table).

After re-reading the text again, I felt that the construct of the sentence was confusing. It was suggesting as if the mean CAT score presented in the text was for “High and Very high impact” group, which was not the intention. Therefore, we decided to make changes to 2 sentences in the baseline characteristic paragraph.

Before change:

Nearly 80% of the Veterans had severe or very severe COPD (GOLD stage 3 or 4). Seventy five percentage of the study population had a high or very high impact of COPD on their daily lives with a mean CAT score of 24.4 ± 7.1.

The new sentences read as below:

Nearly 80% of the Veterans had severe or very severe COPD (GOLD stage 3 or 4) and the impact of COPD in their lives as measured by COPD Assessment Test (mean CAT score 24.8 ± 7.1) was very high. Moreover, 75% of the study population had a high or very high impact of COPD on their daily lives.

The break down for CAT score groups was as follows:

Medium impact group (CAT score >10 to 20)

n=13, mean ± SD, 15.5 ± 3.0

High or very high impact group (CAT score of > 20)

n=39, mean ± SD: 27.9 ± 4.9

Proportional break down of the CAT score provides a good insight into the data. Adding additional piece of data would have cluttered the table more (in our opinion). Therefore, we decided not to add anything more to the table.
