# Supplementary material for: Impact of pulmonary rehabilitation in sleep in COPD patients measured by actigraphy
Source: PLoS One. 2021 Mar 16;16(3):e0248466. doi: 10.1371/journal.pone.0248466 (PMC7963048; doi:10.1371/journal.pone.0248466)
Supplement: Attachment — Submitted filename: Response to the reviewer_3.docx [file pone.0248466.s003.docx]

**PONE-D-20-23683**

**Response to the reviewer**

**Comment from the reviewer:**

Please check carefully again the data in the text and in the tables because some disagreements are still present.

For example- Non-obese pts: 52.3 % in the text and 62.3% in the table.

**Response:**

Thank you very much for spotting the typographical error. The value in the table (62.3%) is accurate. This was crossed check with data output and corrected in the updated manuscript.

We also carefully checked the entire manuscript for other typographical and formatting errors as well as grammatical errors. We found few and have made changes as below:

1) Results/Demographics: “80%” should have been “70%”

Used to read: “Nearly 80% of the Veterans”

Now reads: More than two-thirds (69.5%) of the Veterans

2) “GOLD stages 3 or 4” were changed to “GOLD stages III or IV”
3) Table 1: “%” signs inside the parentheses in the second column were removed since it was already indicated in the first column. For example:

Used to read as: 43 (62.3%)

Now reads: 43 (62.3)

4) Table 1: Minimal cognitive impairment was changed to Mild cognitive impairment since SLUMS does not have minimal cognitive impairment in the scale.

5)	All sentences with “veterans” were changed with “Veterans”

6) Result/Sleep quality and factors affecting sleep:

- Chronic pain prevalence was 52% not 42%. It was cross checked and corrected in the edited manuscript.

7) Results/Effect of PR on COPD related outcomes:

-p value for change in CAT score was <0.0001 not <0.001.

8) Punctuations and grammatical errors were corrected as appropriate.
